# Sectional Anatomy Quiz - IV

**DOI:** 10.22038/AOJNMB.2019.38842.1260

**Published:** 2019

**Authors:** Adil Lathif, Rashid Hashmi

**Affiliations:** Rural Clinical School, University of New South Wales (UNSW), Wagga Wagga, NSW, Australia

**Keywords:** Anatomy, Computed tomography, Heart

## Abstract

In this series we present a quiz about identification of salient and important anatomical landmarks present at a given level on the computed tomography (CT) image. The representative image is followed by further images showing examples of various commonly encountered pathologies that can be seen at this level in clinical practice. Readers are expected to identify highlighted structures in all the images and appreciate how a given abnormality can alter the appearance of normal structures. The aim of this series is to foster understanding and interpretation of the CT component of the single photon emission computed tomography (SPECT) and positron emission tomography (PET) studies help nuclear physicians in interpretation by the nuclear medicine professionals.

## Introduction

***Answer***

The image is through the lower chest at the level of the heart and shows normal appearance of four cardiac chambers at the level of cardiac apex. 

A: Right atrium

B: Right ventricle

C: Left atrium

D: Left ventricle

E: Descending thoracic aorta

F: Oesophagus

Arrows point to the pericardium, dotted lines show normal wall thickness of the lateral wall of the left ventricle (which is normally three times thicker that the wall of the right ventricle) and asterisk points to the inter-ventricular septum.

**Figure 1:**
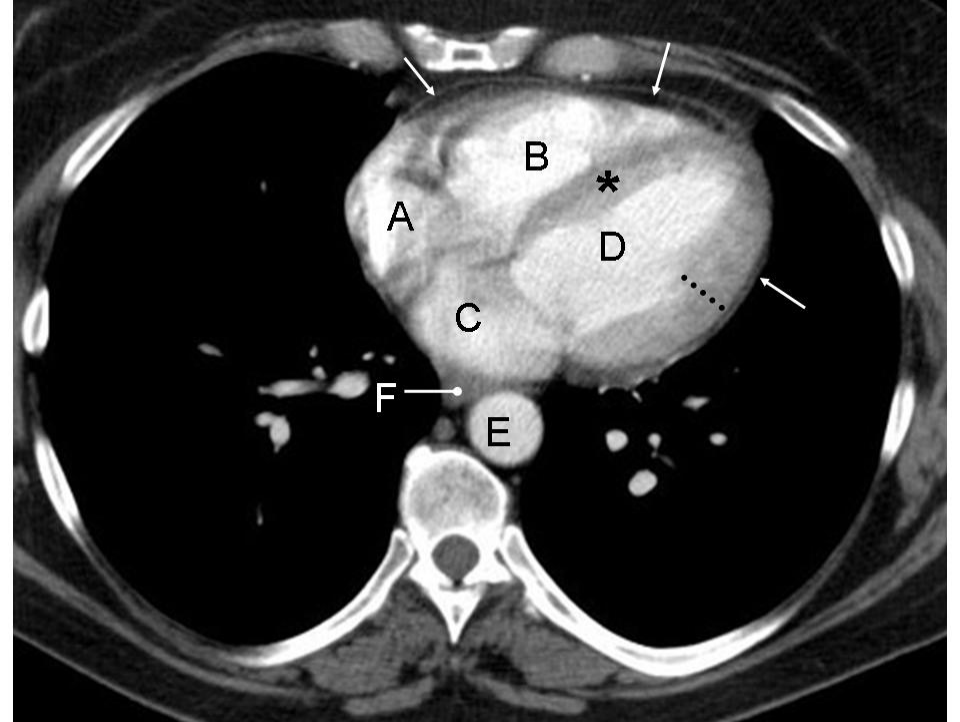
A contrast enhanced axial CT image of the chest of a 45 years old male is shown. Identify the labelled normal anatomical structures

**Figure 2:**
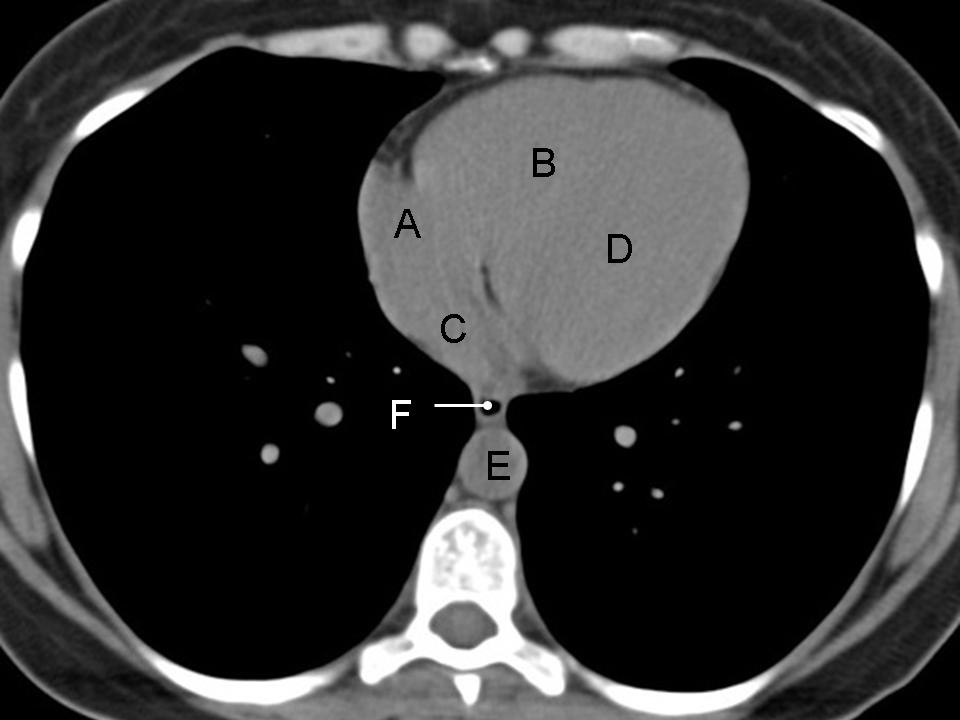
Non-contrast CT of the chest of a 34 years old male shows normal appearance of right atrium (A), right ventricle (B), left atrium (C), left ventricle (D), descending thoracic aorta (E) and oesophagus (F). Note that outlines of individual cardiac chambers and inter-ventricular septum are not clearly delineated on the non-contrast image. Compare the appearance of the oesophagus with that in the Figure 1 to appreciate the difference between its collapsed and air-filled segment

**Figure 3:**
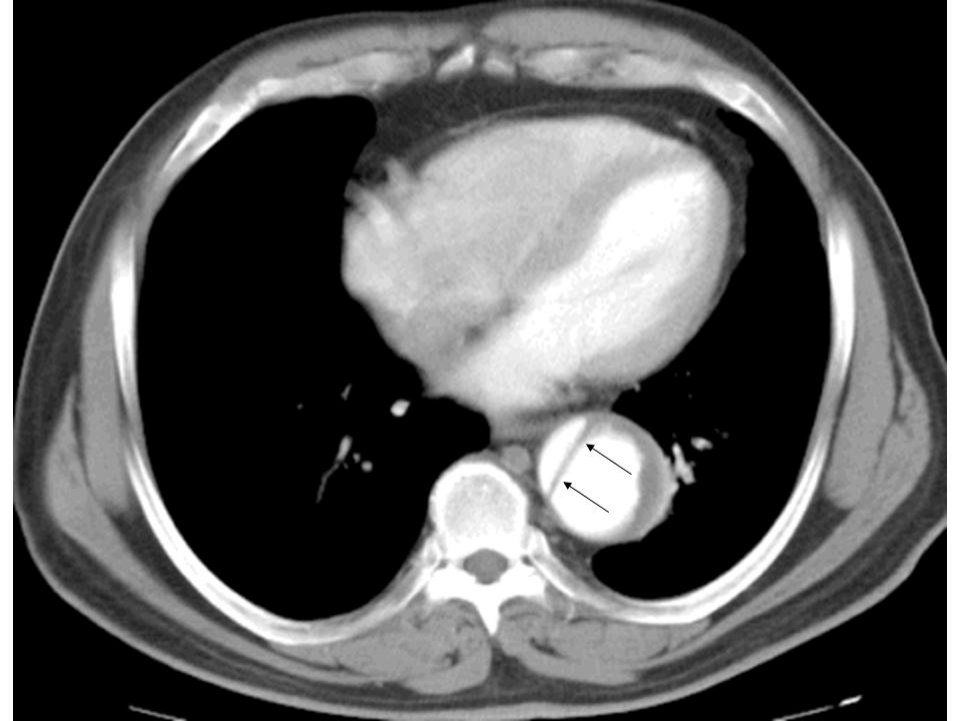
Contrast enhanced CT of the chest obtained in a 67 years old hypertensive male who presented with acute central chest pain shows aortic dissection involving the descending thoracic aorta. Arrows point to the intimal flap. It is important to appreciate that the size and appearance of cardiac chambers on a contrast enhanced image depends on the amount of contrast present in a given chamber at the time of acquisition of the image and is influenced by factors likes volume and speed of injection of the of contrast, time difference between injection of contrast and acquisition of image, cardiac output of a given subject and type of CT scanner

**Figure 4:**
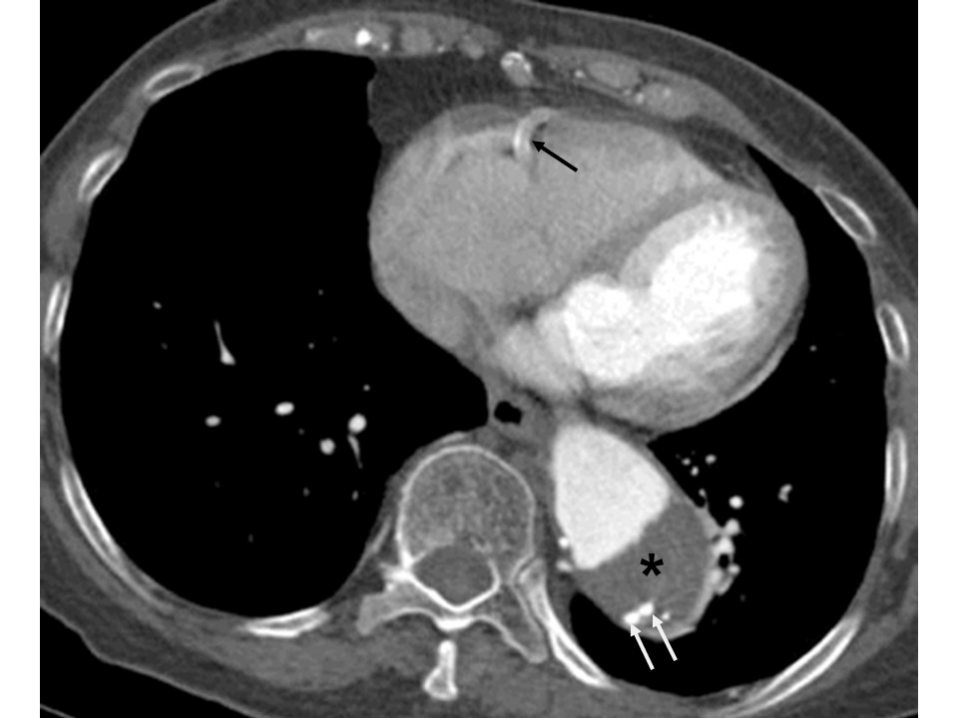
Contrast enhanced CT of the chest of a 73 years old male shows dilatation of the descending thoracic aorta suggesting aneurysm. Asterisk points to an intramural thrombus. Small areas of high attenuation within posteromedial aspect of the thrombus (white arrows) most likely represent bleeding within the thrombus and could be a sign of an imminent rupture necessitating early surgical review. Calcification (black arrow) of the right coronary artery is incidentally noted

**Figure 5:**
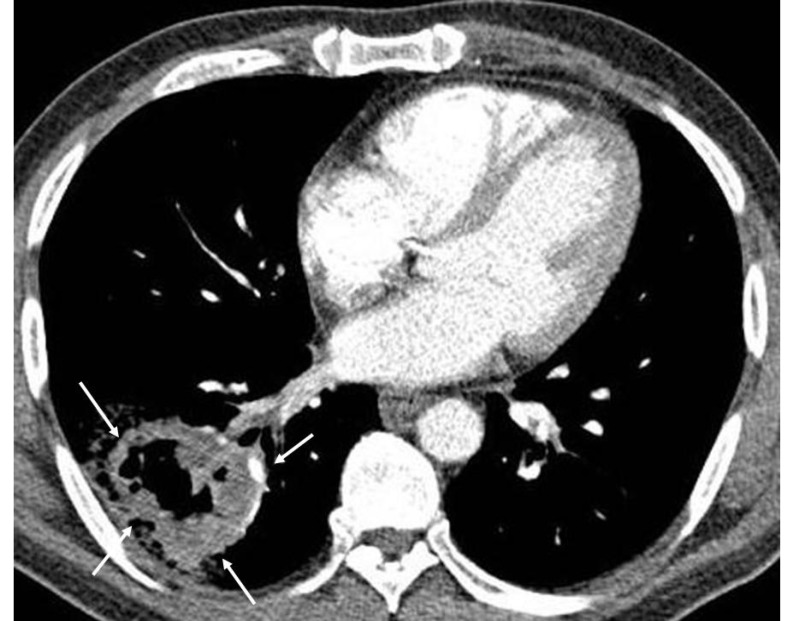
Contrast enhanced CT of a 59 years old male smoker with bronchogenic carcinoma shows a large mass (arrows) located peripherally in the lower lobe of the right lung. Mass has an irregular margin and contains air suggesting presence of necrotic changes. In addition to a malignancy, necrotic pulmonary lesion can also be encountered in the setting of pyogenic, mycobacterial and fungal infection, metastasis, Wegener’s granulomatosis, infarction etc

**Figure 6:**
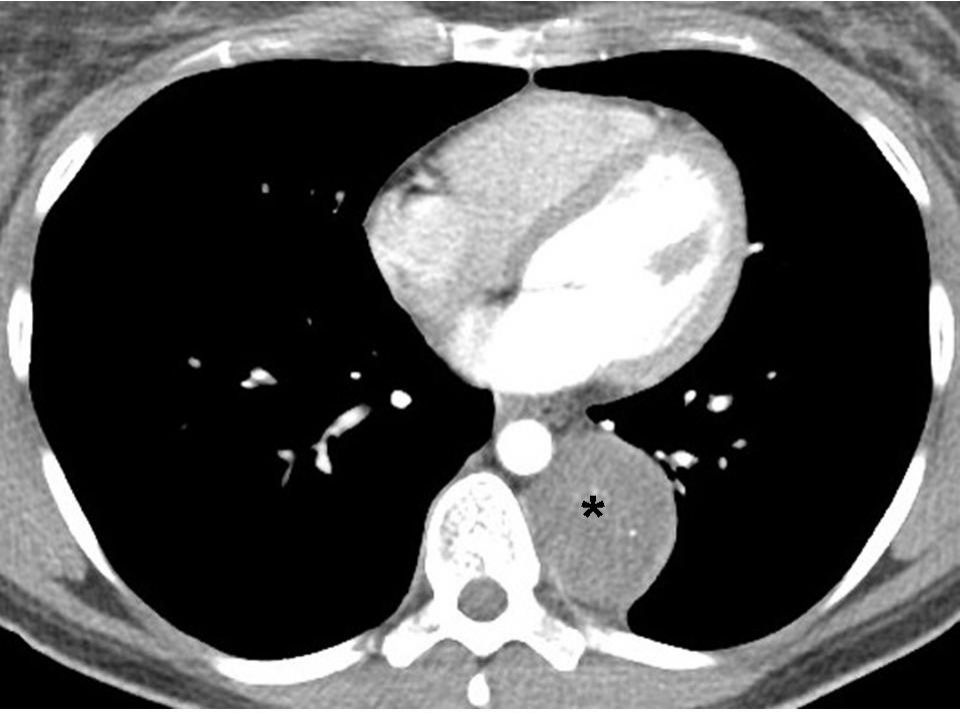
Contrast enhanced CT of a 35 years old female with history of vague chest pain for few months shows a well marginated enhancing mass (asterisk) in posteromedial aspect of the left hemi-thorax in para-vertebral location. The mass forms an obtuse angle with the chest wall suggesting that it is most likely extra-pulmonary in location. Main differential diagnosis of a solid mass in this location is a neurogenic tumor. These tumors can arise either from nerve roots (schwanoma, neurofibroma etc.) or sympathetic ganglia (neuroblastoma, ganglioneuroma, ganglioneuroblastoma etc.)

**Figure 7:**
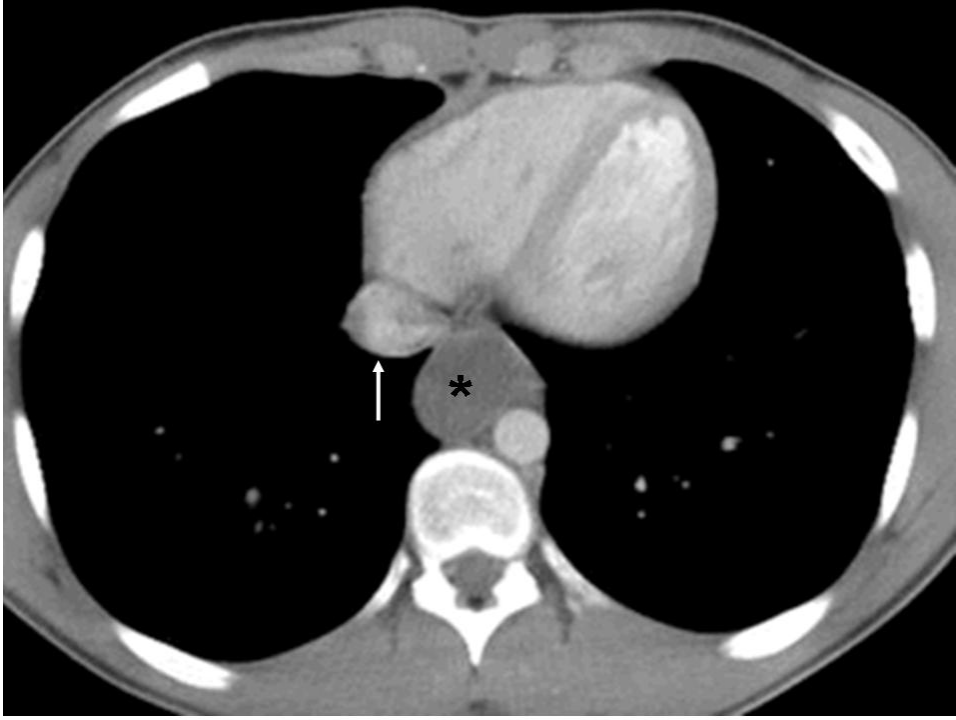
Contrast enhanced CT on a 45 years old male show a low density mass (asterisk) located posterior to the heart and anterior and to the right of the descending thoracic aorta. Oesophagus is not separately identified from the mass. Density of the mass was measured to be 7HU suggesting that it is cystic in nature. Differential considerations of cystic mass in this location are bronchogenic cyst and oesophageal duplication cyst. Arrow points to inferior vena cava as it enters right atrium

**Figure 8:**
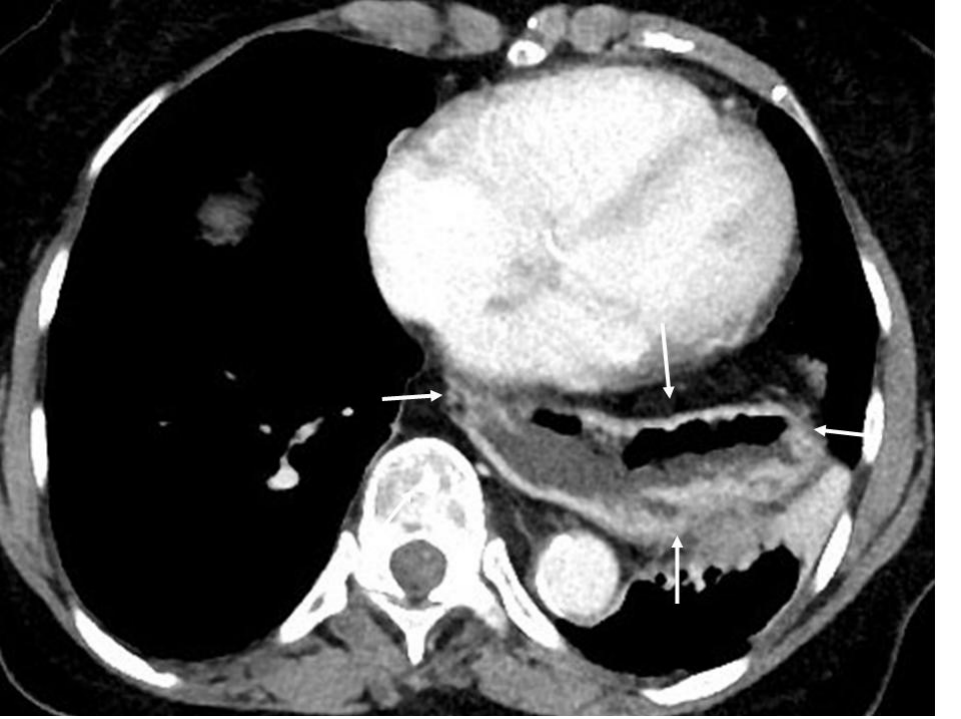
Contrast enhanced CT of the chest of an 84 years old female with complains of chest tightness, regurgitation and dyspepsia for a few years shows a large mixed density mass (arrows) in the left hemi-thorax behind in the heart. The appearance is typical of a hiatus hernia. Density of hiatus hernia is variable but generally contains air and fluid

**Figure 9:**
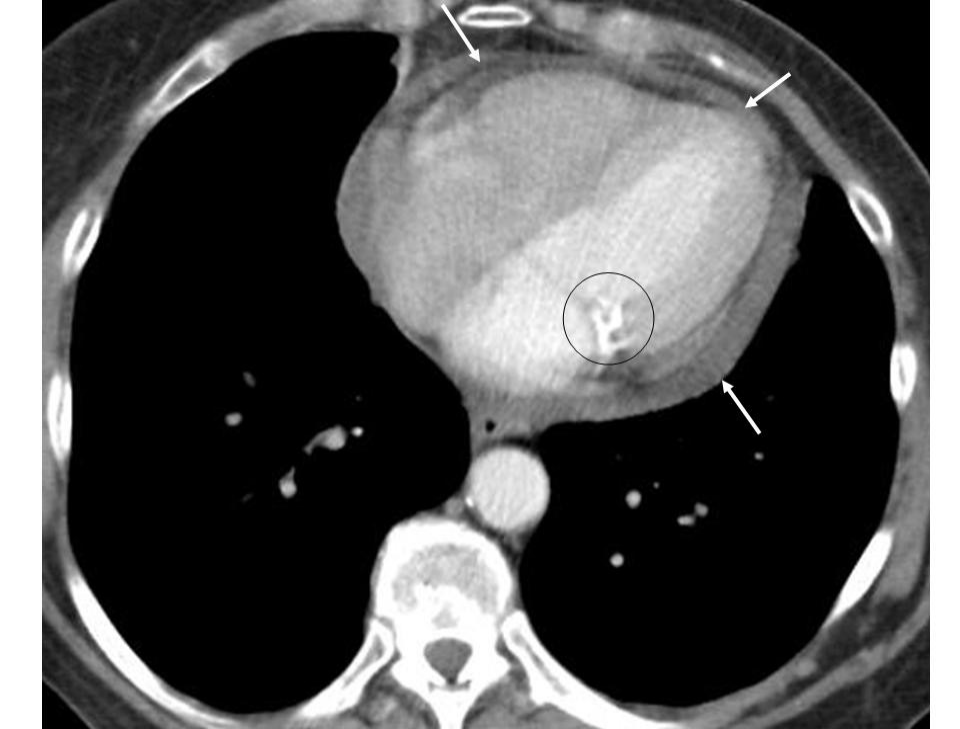
Contrast enhanced chest CT of a 65 years old female shows accumulation of fluid around the heart (arrows) suggesting pericardial effusion. Given that she was a known case of carcinoma breast, pericardial effusion was considered to be malignant in nature. Mitral valve calcification (circle) is noted at the junction of the left atrium and left ventricle

**Points to** **r** **emember**

- On contrast enhanced images, enhancement of cardiac chambers is variable and depends of multiple factors like volume and speed of contrast injection, interval between injection of contrast and acquisition of images, indication for a given study, type of scanner, cardiac output of the patient etc.
- Left ventricle forms the left lateral margin of the heart. At the level of cardiac apex, it is elliptical in shape and its long axis is directed laterally and anteriorly. Though the left ventricular myocardium is nearly uniform in thickness, on axial images due to the obliquity lateral wall appears more thicker than the apical or septal walls.
- Right ventricle is the most anterior of the cardiac chambers and lies anteriorly and to the right of the left ventricle. Its wall is typically thinner than the left ventricular wall. On more superior images right ventricular outflow tract is outlined as a circular density lying anterior and to the left of aorta.
- Interventricular septum is obliquely oriented and is normally convex interiorly towards the right ventricle due to higher left ventricular pressure.
- Right atrium forms the right margin of the heart. Superior and inferior vena cava are seen as discrete oval structures entering in the posterior aspect of the right atrium on more cranial and caudal images respectively.
- The left atrium is the most superior and posterior chamber of the heart and receives blood from the pulmonary veins. It lies posterior, superior, and to the left with respect to the right atrium.
- Pericardium is visible as thin 1-2 mm stripe of soft tissue paralleling the heart. Its visualization is dependent on the amount of epicardial and mediastinal fat that surrounds it.
- Cardiac valves are not identified on standard contrast enhanced CT but their location can be inferred by indentifying the outlines of cardiac chambers. Calcified valves are easily identifiable on non-contrast images.

**Recommendations for Further Reading**

1. O’Brien JP, Srichai MB, Hecht EM, Kim DC, Jacobs JE. Anatomy of the heart at multidetector CT: what the radiologist needs to know. Radiographics. 2007;27(6):1569-82.
2. Faletra F, Pandian N, Ho SY. Anatomy of the heart by multislice computed tomography. New Jersey: John Wiley & Sons; 2009.
3. Ryan S, McNicholas M, Eustace SJ. Anatomy for diagnostic imaging e-book. 3^rd^ ed. New York: Elsevier Health Sciences; 2011.
4. Currie S, Kennish S, Flood K. Essential radiological anatomy for the MRCS. Cambridge: Cambridge University Press; 2009.
5. Moeller T, Reif E. Pocket atlas of sectional anatomy. Computed tomography and magnetic resonance imaging. Thorax, abdomen, and pelvis. Stuttgart: Thieme; 2001.

